# Future agricultural systems and the role of digitalization for achieving sustainability goals. A review

**DOI:** 10.1007/s13593-022-00792-6

**Published:** 2022-07-06

**Authors:** Joseph MacPherson, Ariane Voglhuber-Slavinsky, Mathias Olbrisch, Philipp Schöbel, Ewa Dönitz, Ioanna Mouratiadou, Katharina Helming

**Affiliations:** 1 Leibniz Centre for Agricultural Landscape Research (ZALF), Eberswalder Straße 84, 15374 Müncheberg, Germany; 2 Fraunhofer Institute for Systems and Innovation Research (ISI), Breslauer Straße, 4876139 Karlsruhe, Germany; 3 Chair of Public Law, Administrative, European, Environmental, Agricultural and Food Law, Prof. Dr. Ines Härtel, European University Viadrina Frankfurt (Oder) | Research Center for Digital Law, Große Scharrnstraße 59, 15230 Frankfurt (Oder), Germany; 4 ISARA Lyon, 23 rue Jean Baldassini, 69364 Lyon, France; 5 Faculty of Landscape Management and Nature Conservation, University for Sustainable Development (HNEE), Schickler Straße 5, 16225 Eberswalde, Germany

**Keywords:** Digital agriculture, Sustainability, Policy, Foresight, Agri-digital law

## Abstract

By leveraging a wide range of novel, data-driven technologies for agricultural production and agri-food value chains, digital agriculture presents potential enhancements to sustainability across food systems. Accordingly, digital agriculture has received considerable attention in policy in recent years, with emphasis mostly placed on the potential of digital agriculture to improve efficiency, productivity and food security, and less attention given to how digitalization may impact other principles of sustainable development, such as biodiversity conservation, soil protection, and human health, for example. Here, we review high-level policy and law in the German and European context to highlight a number of important institutional, societal, and legal preconditions for leveraging digital agriculture to achieve diverse sustainability targets. Additionally, we combine foresight analysis with our review to reflect on how future frame conditions influencing agricultural digitalization and sustainability could conceivably arise. The major points are the following: (1) some polices consider the benefits of digital agriculture, although only to a limited extent and mostly in terms of resource use efficiency; (2) law as it applies to digital agriculture is emerging but is highly fragmented; and (3) the adoption of digital agriculture and if it is used to enhance sustainability will be dependent on future data ownership regimes.

**Supplementary Information:**

The online version contains supplementary material available at 10.1007/s13593-022-00792-6.

## Contents

- 1. Introduction
- 2. Methodology
- 3. Connections to digital agriculture and sustainability principles in policy
- 3.1 Digital agriculture in policy
- 3.2 Cross-cutting sustainability principles
- 3.2.1 Biomass production
- 3.2.2 Climate change mitigation and adaptation
- 3.2.3 Biodiversity conservation
- 3.2.4 Soil health
- 3.2.5 Health and nutrition
- 3.3 Key digital technologies for achieving sustainability principles
- 4. Foresight and its implications for sustainability principles and agri-digital law
- 4.1. Description of future scenarios
- 4.1.1. Scenario 1: Environmental Protection by Global High Tech and Regulation; Globalized world, government regulation and harmonization
- 4.1.2. Scenario 2: Environmental Protection by Local Food Circles and Qualitative Growth; Decentralization, diversity and sustainability
- 4.1.3. Scenario 3: Event Consumption by Face-to-Face Interaction in Local Food Circles; Consumption and direct communication
- 4.1.4. Scenario 4: Reduced Consumption and De-growth by Necessity; Growing retail business, no transparence and global food system
- 4.2 Combination of scenarios and key technological areas
- 4.3 Convergence of sustainability principles and scenarios
- 5. Agri-Digital Law
- 6. Discussion
- 7. Conclusion
- Declarations
- Literature Cited

## Introduction

Digitalization is a rapidly growing trend within agriculture. Digital agriculture, or “Smart Farming,” is characterized by the use of precision and data-driven technologies to assist farmers with real-time and site-specific decision making (Wolfert et al. 2017; Rose and Chilvers 2018; Weersink et al. 2018). It leverages technologies including the Internet of Things (IoT), sensors, drones, robotics, cloud computing, artificial intelligence (AI), decision support software (DSS), and blockchain, for example, to optimize agricultural production processes (Walter et al. 2017; Kamilaris et al. 2017), value chains (Poppe et al. 2013, Smith 2020), international trade (Jouanjean 2019), agricultural systems (Basso and Antle 2020), and governance systems (Ehlers et al. 2021).

By and large, digital agriculture is viewed as a promising means for sustainably boosting food production to feed a growing world population (Foley et al. 2011; Shepherd et al. 2020). Along with improving agricultural productivity, digitalization could provide a diverse range of benefits to the environment and society. For instance, digital agriculture could help alleviate pressures on scarce resources (Wolfert et al. 2017), improve food safety through increased traceability (Walter et al. 2017), as well as combat climate change (Balafoutis et al. 2017). Other potential benefits of agricultural digitalization include the creation of new types of high-skilled job opportunities (Rotz et al. 2019b), fostering global agricultural markets (Jouanjean 2019), as well as improvements to animal welfare (Dawkins 2017).

Due to the relative novelty of digital agriculture, there is still a considerable amount of uncertainty surrounding its impact on sustainability (Klerkx and Rose 2020). Skeptics have warned that digitalization could perpetuate *status*-*quo* economic modes of production (Bronson and Knezevic 2016), while raising concerns about the ownership, privacy and sovereignty of data, and how this could reinforce concentrations of power among large ag-tech service providers (Rotz et al. 2019a; Clapp and Ruder 2020). Additionally, automation could lead to displacement of certain types of low-skilled jobs in the agri-food sector (Carolan 2020), or could lead to “algorithm governance” where farmers lose their autonomy to manage their own farms (Henman 2020). Lastly, the electricity demand required to power the infrastructure underpinning digital technologies (e.g., servers) and potential greenhouse gas emissions therein could produce spillovers and deserves further exploration (Leroux 2020).

Nevertheless, the potential benefits of digital agriculture have garnered attention in policy circles and are increasingly included, albeit as a side topic, in high-level policy strategies. To date, no study has tried to summarize this development, with the exception of Lajoie-O’Malley et al. (2020). Their findings pointed out that visions of digitalization as articulated by international institutions such as the World Bank, Organization for Economic Co-operation and Development (OCED), and Food and Agricultural Organization (FAO) focus primarily on reducing food shortages through agricultural intensification, while largely ignoring environmental concerns, such as the provision of ecosystem services.

Principles and agreements outlined in high-level policy strategies (soft law) play a crucial role in determining the frame conditions for technological innovation and adoption through shaping public discourse, directing public funding for research and development, as well as setting subsidies and regulations (hard law). In this respect, policy can have a strong influence on the future of digital agriculture. Therefore, it is necessary to take stock of how current policy strategies consider agricultural digitalization and, going further, investigate how digitalization can implicitly support broader sustainability goals. Additionally, given the undetermined future of digital agriculture, studies are needed that plot potential trajectories of societal trends to assess how it may affect sustainability (Klerkx and Rose 2020). Finally, equal consideration needs to be given to the evolving legal landscape surrounding digital agriculture, as this will play an important guiding role in the digital transformation of agriculture, as well (Härtel 2019, 2020a).

It is worth noting that institutional, regulatory, and socio-technical conditions vary across countries, cultures, and scales, meaning the frame conditions for digital agriculture and the way it is instrumentalized may also vary. For example, in terms of policy, the USDA Agricultural Innovation Agenda (United States Department of Agriculture (USDA) 2020) and the European Commission Farm to Fork (F2F) Strategy (European Commission 2020a) both acknowledge the importance of sustainability and reducing the environmental footprint of the agricultural sector. While both policies incorporate digital agriculture within their strategies, the former embraces a productivist paradigm with no restrictions on the input of pesticides or fertilizers, while the latter focuses on resource use efficiency improvements by setting targets to starkly reduce agrochemicals inputs. These two fundamentally different approaches could lead to very different manifestations of digital agriculture in the future. In this regard, studies are needed that account for these context-specific factors when assessing possible developments of agricultural digitalization.

To these ends, this research is motivated by the following questions: (i) how is digital agriculture currently embedded in preeminent global, EU, and German policy, and what links can be drawn between digital agriculture technologies and to wider sustainability principles outlined in these policies; (ii) how could future trends in the agri-food sector influence the adoption and use of digital technologies; and (iii) how does the current legal setting surrounding digital technologies impact agriculture? The results of this study are meant to highlight a number of important institutional, societal, and legal preconditions for leveraging the potential of digitalization to align agricultural production with sustainable development targets. Further, our research offers a novel example of transdisciplinary research by combining policy, foresight and legal analyses.

**Fig. 1:**
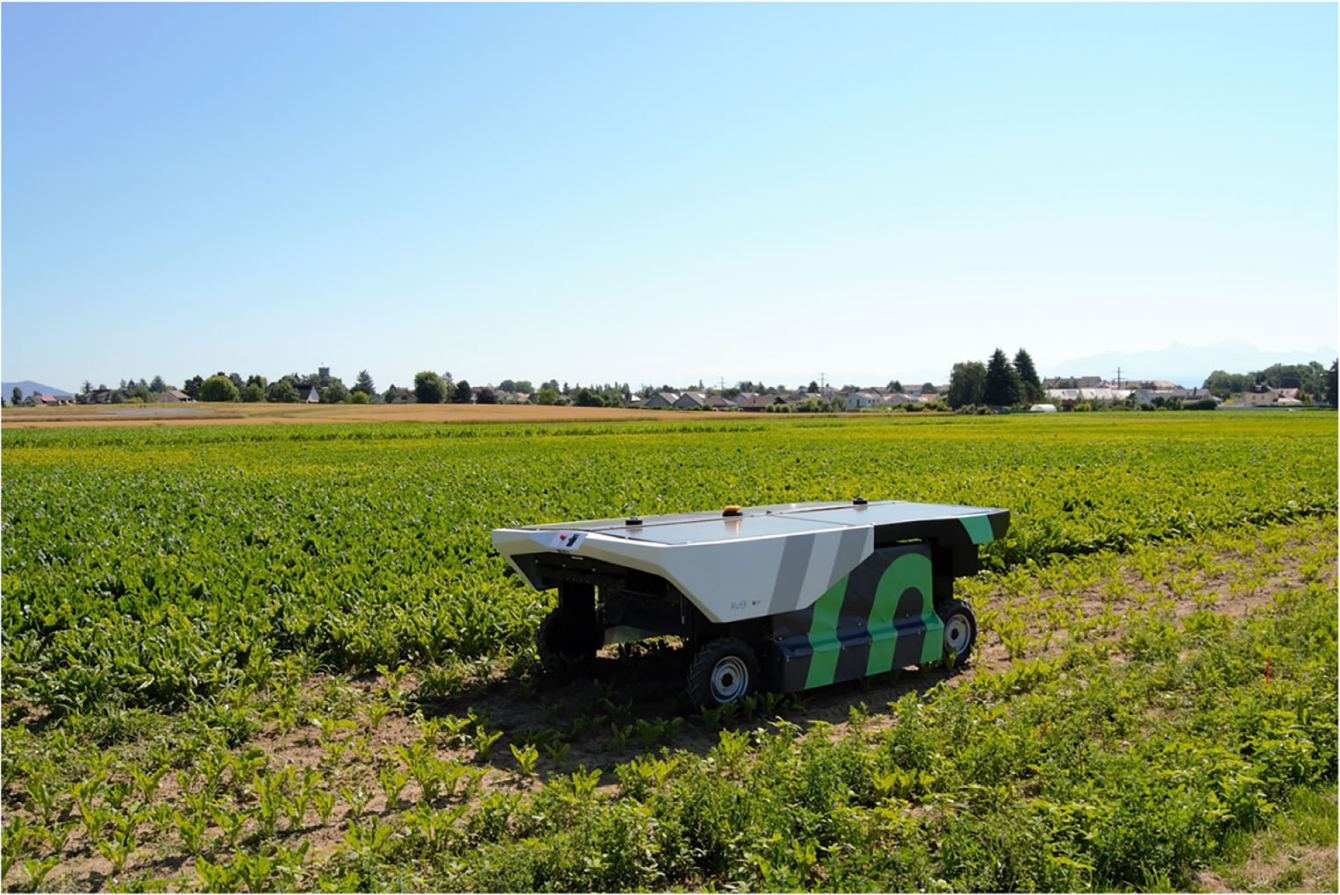
Autonomous weeding machine (AVO) from ecoRobotix. Photo available for download from https://ecorobotix.com/en/contact

The paper is organized as follows. Section 2 provides an overview of the methodologies employed in the policy, foresight and legal analyses. Section 3 reviews agriculture-related goals of several preeminent policy strategies at the global, European, and German national level, paying particular attention to how digitalization is articulated within each strategy, as well as drawing links between agriculture-related goals and key enabling technologies from the literature. Section 4 concerns the foresight analysis, describing future frame conditions of four different scenarios, and analyzing how they affect hotspots of agricultural digitalization and the achievement of sustainability principles. Section 4 reviews current agri-digital law across multiple governance levels. Section 5 synthesizes and discusses the results of the proceeding sections which is followed by a conclusion in Section 6 (Fig. 1).

## Methodology

In Section 3, we reviewed the documents of seven preeminent sustainability policies spanning German, European, and global policy levels. These policies were selected based on the judgment of the authors that they are highly relevant in regard to their influence on agricultural sustainability and, in general, guiding the development of lower-level policies and regulations. Germany was chosen as a focal point for this study due to its relatively advanced agri-food sector and for its standing as a notable leader in sustainability and bioeconomy policy. In the first step of the policy review process, policy documents were scanned for agriculture-related goals as well as links to digital agriculture (Section 3.1). Then, agriculture-related goals were inductively sorted into clusters based on cross-cutting sustainability principles that emerged from the policies (Section 3.2). Finally, connections were drawn between the agriculture-related goals and key digital technologies (Section 3.3). Examples of key technologies and their potential applications were taken from the literature (Wolfert et al. 2017; Lieder and Schröter-Schlaack 2021; Weersink et al. 2018).

In Section 4, we present four different scenarios, providing insights into how the framework conditions for agriculture in 2035 in Germany might look like, including what the effects on natural resources could be as well as what role digital decision support systems can play for farmers in this context (Dönitz et al. 2020). The framework conditions for German agriculture are subject to constant change. Yet despite these uncertainties, we can still make assumptions about probable future developments. Indeed, political, economic, societal, ecological, and technological developments must be included to create robust solutions and scenarios assist to deal with the high complexity of these interacting factors. Based on a complex network of relevant factors, the scenarios present a description of possible situations in the future. The scenario method is an established and proven instrument within the foresight methods for addressing uncertainties (Gabriel et al. 2016; Dönitz and Schirrmeister 2013; Godet 2001; van Notten et al. 2003).

Across the scenarios, we identified the most influential factors for the topic of digitalization in agriculture. Key factors such as “Information flow along the value chain and acceptance of service platforms” and “Diffusion of new technologies in primary production” have a strong influence on all other and are highly relevant for digitalization in agriculture. We presented the respective future assumptions of these two factors per scenario and further combined the information with the key technological areas, while also highlighting respective legal implication (Section 4.2). We then explore how these scenarios converge with sustainability principles identified in the policy analysis (Section 4.3).

In Section 5, we review the current state of law surrounding digital agriculture, which is situated in a legal multi-level system, at the European and German national level. We outlined requirements for a consistent legal framework as an enabler for the digital transformation of agriculture by analyzing legal implications for the policies and the foresight scenarios.

**Fig. 2:**
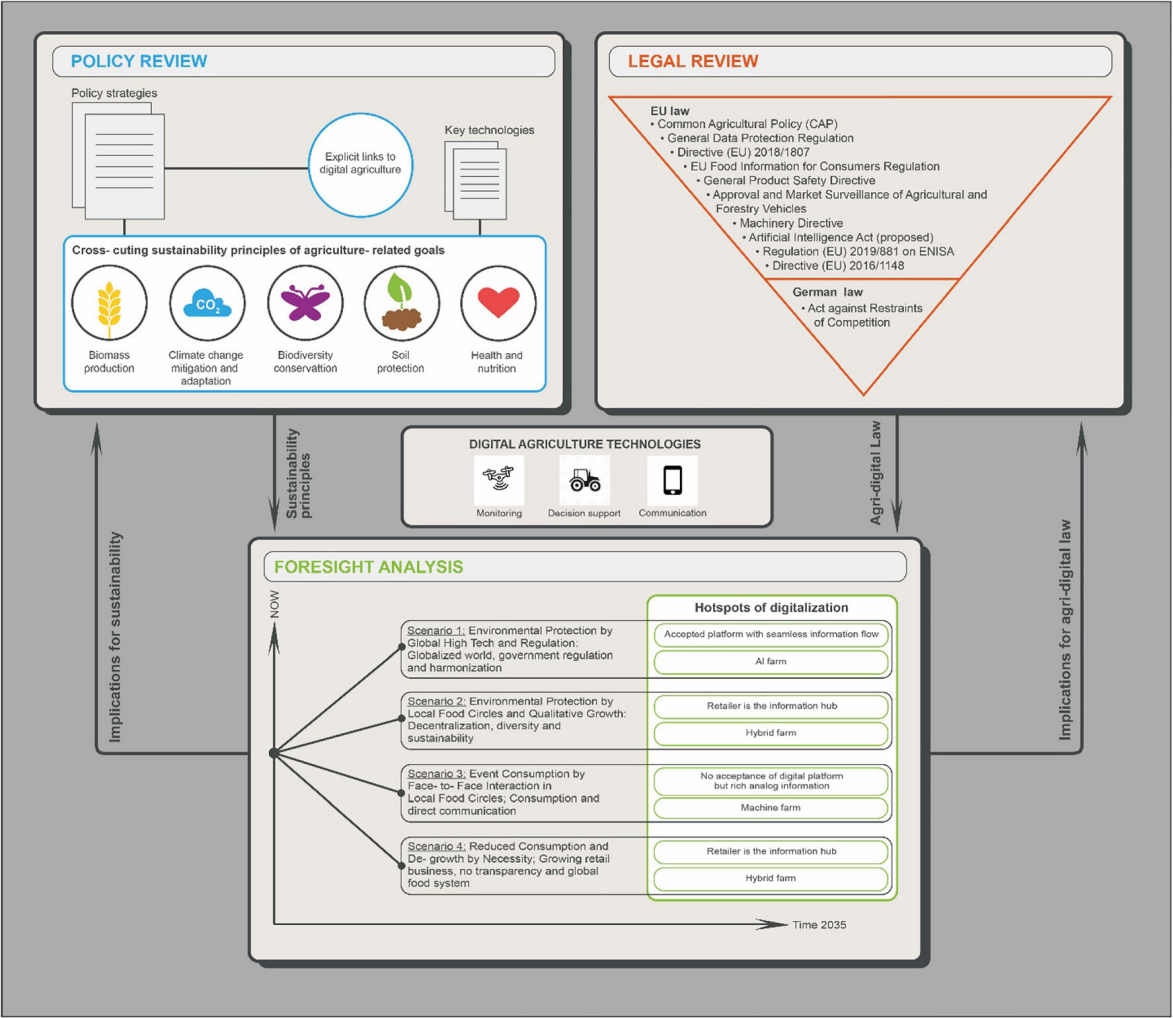
An overview of the methods and linkages of the different sections. Implications of digital agriculture as found in the policy and legal reviews are explored through scenarios in the foresight analysis to reflect on future sustainability

To structure our research and provide linkages between the policy, foresight, and legal analyses, we focus on the contribution of agricultural digital technologies to enhance the sustainability of agricultural systems via improved *monitoring, decision support*, *and communication* as suggested by Mouratiadou et al. (2021) (see Table 1). For an overview of the methodology, see Fig. 2.

**Table 1:** Functions of digital technologies for sustainable agriculture (adopted from Mouratiadou et al. 2021)

Function	Description
Monitoring	Effective and transparent monitoring of biodiversity and ecosystem service provision, facilitating the understanding of cause-effect relationships in agroecosystems and the establishment of result-oriented policy measures
Decision support	Improved agricultural decision support, for multifunctional diversified agricultural landscapes to consolidate diverse targets on yields, ecosystem services, biodiversity, and deliver resource use efficiency improvements
Communication	Enhanced communication between stakeholders and land use actors, enabling information exchange on societal demands on biodiversity and ecosystem services along the value chain and reducing conflicts on the future use of agricultural land

## Connections to digital agriculture and sustainability principles in policy

A review of high-level policy strategies revealed a multitude of agricultural-related sustainability goals and several links to digital agriculture (see Table 2 for overview of policies included in the review). In relation to the former, we were able to cluster goals according to five sustainability principles, which emerged as cross-cutting themes in the policies. In the following sub-sections, explicit links to digitalization as found within the reviewed policy documents are presented (Section 3.1). Agriculture-related goals as found within the policy documents are outlined according to the following cross-cutting sustainability principles: biomass production (Section 3.2.1), climate change mitigation and adaption (Section 3.2.2), biodiversity conservation (Section 3.2.3), soil health (Section 3.2.4), and health and nutrition (Section 3.2.5). Finally, we draw links between agriculture-related goals and key enabling digital technologies (Section 3.3).

**Table 2:** Overview of policies included in the review

Policy strategy	Publisher	Governance level	Reference
Paris Agreement	United Nations Framework Convention on Climate Change (UNFCCC)	Global	UNFCCC Secretariat 2019
Sustainable Development Goals (SDGs)	United Nations (UN)	Global	United Nations 2016
Farm to Fork (F2F) Strategy of the European Green Deal	European Commission	Europe	European Commission 2020a
Climate Action Plan 2050	Federal Ministry for the Environment, Nature Conservation, Building and Nuclear Safety (BMU)	National (Germany)	Deutsche Bundesregierung 2016
National Sustainable Development	German Federal Government	National (Germany)	Deutsche Bundesregierung 2017 & 2018
2035 Arable Farming Strategy	Federal Ministry of Food and Agriculture (BMEL)	National (Germany)	Bundesministerium für Ernährung und Landwirtschaft 2018
National Bioeconomy Strategy	Federal Ministry of Food and Agriculture (BMEL)	National (Germany)	Bundesministerium für Ernährung und Landwirtschaft (BMEL) 2020

### *Digital agriculture in policy*

To date, there is no comprehensive strategy dedicated specifically to digital agriculture at the global, European, or German policy level. However, digitalization is often considered by policy as a driver, or means, toward achieving certain sustainability goals. Three of the reviewed policies refer explicitly to digital agriculture (the F2F Strategy, the German 2035 Arable Farming Strategy, and the German National Bioeconomy Strategy), which is summarized in Table 3. The remaining policies of this review (e.g., the Paris Agreement, the SDGs, the National Climate Action Plan 2050, and the German Sustainability Strategy) do not explicitly consider agricultural digitalization within their documents.

**Table 3:** Policy strategies and explicit links to digital agriculture

Policy strategy	Links to digital agriculture
F2F Strategy	• Use climate data to improve adaptation to climate change • Increase resource use efficiency via precision technologies • Provide more information to consumers using digital solutions • Secure Common Agricultural Policy funds toward fostering digital innovation • Increase access to high-speed broadband internet to rural areas to mainstream adoption of use of precision agriculture and artificial intelligence (satellites) • Broaden agricultural databases i.e. Farm accountancy data network (FADN) • Create common European agricultural data space
2035 Arable Farming Strategy	• Increase mobile network coverage • Establish quality control body for digital applications • Develop innovative digital technologies for soil tillage, fertilization and plant protection to promote healthy soils • Make technology available for small and medium-sized farms, as well as for multi-farm use • Create statutory framework conditions for the use of digital technologies • Implement nationwide coverage of real-time kinematic -GPS and ensure access to public data for farmers • Establish test sites for new technologies throughout Germany • Review preconditions for establishing ‘data sovereignty’
National Bioeconomy Strategy	• Improve understanding of systemic modeling • Foster data harmonization • Improve data management systems • Advance user interfaces • Implement standards • Use big data for quantification of the impacts of bioeconomy measures to overall economy

The F2F Strategy acknowledges the importance of digitalization for making more efficient use of agricultural inputs, as well as making better use of climate and environmental data for improving the resilience of food systems to the impacts of climate change (European Commission 2020a). The Strategy also aims to exploit the potential of digitalization in value chains using product tracking to provide consumers with more information regarding how their food is produced, thereby promoting healthier and “greener” food choices. The increase of the availability of high-speed broadband internet to rural areas throughout the EU is also in focus so that farmers can better capitalize on digital technologies, including AI and precision techniques that lead to better soil management. Additionally, the F2F Strategy intends to expand the Farm Accountancy Data Network (FADN) for monitoring as well as creating a common European agricultural data space for fostering interoperability of data.

At the German national level, digitalization is one of the twelve “action areas” included in the 2035 Arable Farming Strategy (Bundesministerium für Ernährung und Landwirtschaft (BMEL) 2019). The Strategy identifies mobile phone and GPS coverage as preconditions to facilitate the use of existing technologies and the development of new resource efficient approaches. The Strategy outlines seven digitalization-related measures: (1) establish an independent “quality control body” for assessing digital applications; (2) improve soil health through developing innovative digital technologies for soil tillage, fertilization, and plant protection; (3) promote digital technology for small and medium-sized farms, as well as for multi-farm use; (4) create statutory framework conditions for the use of digitalization; (5) implement nationwide coverage of real-time kinematic GPS and ensure access to public data for farmers; (6) establish test sites throughout Germany; and (7) review preconditions to establishing “data sovereignty” of farmers.

Germany’s National Bioeconomy Strategy underscores the potential of combining digitalization and simulations to improve understanding of systemic modeling (Bundesministerium für Ernährung und Landwirtschaft (BMEL) 2020). Systemic modeling, as the Strategy advocates, should be used in “impact assessment, prediction and the targeted design of efficient and tailor-made bio-based processes” (p.30, ibid.). In conjunction, measures that involve monitoring and control of bio-technological processes, smart sensor technology, AI, automation, miniaturization, parallelization of process steps, and high-throughput analyses are prioritized under the Strategy. The Strategy also identifies the necessity of data harmonization, data management systems, advancement of interfaces, and development and implementation of standards as preconditions to the successful integration of digitalization in the future bioeconomy. Lastly, increased digitalization and “big-data” analysis will enable the quantification of the impacts of bioeconomy measures and their contribution to the overall economy.

### *Cross-cutting sustainability principles*

#### *Biomass production*

The primary task of agriculture is to produce biomass for food, energy, and materials. In the SDGs, food production is addressed by SDG 2 (Zero Hunger) with the objective to increase agricultural productivity and incomes (Target 2.03) (United Nations 2016). In Europe and Germany, food production is relatively high, so biomass production, as it relates to food security, is not perceived as a crucial sustainability issue. However, in the F2F Strategy, food production within Europe is addressed in terms of promoting resilience of food systems against shocks and crises, such as the recent COVID-19 pandemic (European Commission 2020a). At the national level, Germany acknowledges its contribution to producing food for the global food system i.e. ‘world food basket’ (Deutsche Bundesregierung 2017, 2018).

Biomass production also plays a central role in the bio-economy by providing a resource base for the production of bio-fuels and bio-materials. As part of the SDGs, Target 7.2 “increase substantially the share of renewable energy in the global energy mix,” can be indirectly linked to the production of biomass for bio-fuels (United Nations 2016). At the European level, the F2F Strategy proposes advancements in the circular, bio-based economy as part of a holistic strategy of the European Green Deal to create a carbon-neutral EU by the second half of the century (European Commission 2020a). Specifically, the Strategy encourages the creation of bio-refineries to produce bio-fertilizers, protein feed, bioenergy, and bio-chemicals. In addition, farms are to reduce methane emission by investing in anaerobic digesters for biogas production from agricultural wastes and residues.

Germany is noteworthy for its history as a leader in advancing bioeconomy policy. In 2010, Germany established the National Research Strategy “BioEconomy 2030” (Bundesministerium für Bildung und Forschung (BMBF) 2010), which focused on building the knowledge base for the bioeconomy by providing funding for public and private research for the development of bioeconomy innovations. In 2013, Germany adopted the National Policy Strategy for the Bioeconomy (Bundesministerium für Ernährung und Landwirtschaft (BMEL) 2014), which set out wide sweeping goals for a creating a sustainable bioeconomy. As of 2020, Germany published the new National Bioeconomy Strategy, building on previous policy strategies and laying out guidelines, strategic goals, and implementation objectives for the funding of research and creation of a policy framework (Bundesministerium für Ernährung und Landwirtschaft (BMEL) 2020). Of the six strategic goals laid out in the Strategy, two share a strong connection to agriculture production, namely “enhance and apply biological knowledge” and “establish a sustainable raw material base for industry.” These goals correspond to measures that will fund research in areas that model biological systems, develop novel production organisms, and sustainably generate biogenic resources. In relation to the latter, the implementation of concrete measures for smart farming, organic farming, and vertical farming are to be prioritized.

#### *Climate change mitigation and adaptation*

From a global perspective, the Paris Agreement of the United Nations Framework Convention on Climate Change (UNFCCC) and the United Nations Sustainable Development Goals (SDGs) represent the preeminent strategies addressing climate change mitigation and adaptation. Under the Paris Agreement, global average temperature is to be kept under a 2 °C rise above pre-industrial levels and nations are required to outline their own intended nationally determined contributions (INDC) toward limiting emissions. As of 2016, 74% of the countries who have signed the Agreement have included measures to limit emission from the agriculture sector as part of their INDCs (UNFCCC Secretariat 2019). Similarly, SDGs aims to reduce the impacts of climate change through mitigation and adaptation (SDG 13: Climate Change), where countries are to strengthen resilience and adaptive capacity of climate change (Target 13.1), as well as implement climate change mitigation measures (Target 13.2) by developing INDCs and national adaption strategies (United Nations 2016).

At the European level, the F2F Strategy aims to limit agricultural GHG emissions by focusing mainly on the livestock sector (European Commission 2020a). Measures to limit these emissions include advancing innovative feed additives and reducing carbon “leakages” from feed imports by promoting EU-grown plant proteins. The Strategy also identifies the potential of agriculture soils to sequester carbon and advocates that farmers should be provided economic incentives for carbon sequestering practices (i.e., carbon farming) through the Common Agricultural Policy and carbon markets.

Within Germany, the National Climate Action Plan 2050 (Deutsche Bundesregierung 2016) sets out to achieve GHG neutrality by the second half of the century. Under the Plan, agriculture should emit no more than 58–61 million tons of CO_2_-equivelants per year by 2030, equating to a 31–34% reduction from 1990 by 2030. Emission reductions in agriculture are to be met primarily by limiting nitrous oxide (N20) emissions from fertilizers and expanding the share of land under organic farming. In relation to the former, nitrogen surpluses are not to exceed 70 kg N/ha by 2028–2032, which is to be achieved through a stricter enforcement of the German Fertilization Ordinance (Düngeverordnung vom 26. Mai 2017 (BGBl. I S. 1305)) and by promoting need-based fertilization using variable-rate technologies. Additionally, the Arable Farming Strategy 2035 acknowledges the importance of reducing nitrous oxide emissions from fertilizers to mitigate GHG emissions (Bundesministerium für Ernährung und Landwirtschaft (BMEL) 2019).

#### *Biodiversity conservation*

Conserving biodiversity and ecosystem integrity is an integral part of attaining the SDGs (United Nations 2016). SDG 2 (Zero Hunger), for example, recognizes the significance of maintaining terrestrial and freshwater ecosystems (target 2.4) and genetic diversity (target 2.5) as the basis for sustainably producing enough food. In concordance with Biodiversity Strategy for 2035 (European Commission 2020c) of the European Green Deal, the F2F Strategy acknowledges the impacts of agriculture intensification on biodiversity. The Strategy identifies the use of chemical pesticides, excess nutrients from fertilizer, and lack of livestock diversity as the primary factors driving agriculture-related biodiversity decline. Under the Strategy, the use of pesticides is to be reduced 50% by 2030 by promoting integrated pest management strategies. In line with the EU Nitrates Directive 91/676/EEC, excess nutrients from fertilizers are to be reduced by 20% by 2030 through precision application methods and low-input farming, thus reducing environmental impacts to biodiversity in water bodies (European Commission 2020a).

Germany’s National Sustainable Development Strategy strives to protect biodiversity and strengthen implementation of the National Strategy for Biological Diversity through achieving 65 targets/indicators, related to increasing diversity and landscape quality, promoting organic farming and reducing agricultural inputs, such as nitrogen- and phosphorous-based fertilizers (Deutsche Bundesregierung 2017, 2018). In the 2035 Arable Farming Strategy, protecting biodiversity is an overarching topic, bridging multiple goals within the strategy regarding soil fertility, crop diversity and rotation, nitrogen surpluses, and plant protection (Bundesministerium für Ernährung und Landwirtschaft 2018). As one of the eight production-areas of action, “Biodiversity” encompasses the halt of species decline, promoting habitat connectivity at the landscape level, establishing regional goals and associated monitoring mechanisms, as well as evaluating economic ramifications of changes in land use to promote biodiversity.

#### *Soil health*

In relation to the SDGs, the importance of soil is formulated in Target 15.3: “restore degraded land and soil, as well as strive for a world that is land degradation neutral by 2030” and Target 2.4 in regard to utilizing agricultural production methods that improve land and soil quality (United Nations 2016). The protection of soil is interwoven with several other primary goals in the F2F Strategy. For example, goals to drastically reduce the use of chemical pesticides and excess nutrients will mitigate the pollution of soil (i.e., fertilizers use should be reduced by at least 20% by 2030 without compromising soil fertility) (European Commission 2020a).

In Germany, the National Bioeconomy Strategy recognizes the importance of developing a bioeconomy that is environmentally sustainable in terms of soil fertility and preserving soil functions, emphasizing the need for a systemic and location-specific approach for the production of biogenic material (Bundesministerium für Ernährung und Landwirtschaft (BMEL) 2020). Among soil-related goals in Germany’s 2035 Arable Farming Strategy, soil fertility and soil biodiversity should be improved, erosion and compaction reduced, humus content should be kept stable through admixture, and land take by non-agricultural usage is to be reduced to under 30 ha per day and net zero by 2050 (Bundesministerium für Ernährung und Landwirtschaft 2018).

#### *Health and nutrition*

On the global level, food security and adequate nutrition are concerns for a large part of the world’s population (FAO, IFAD, UNICEF, WFP and WHO 2020). This is addressed by the UN SDGs by SDG 2 (Zero Hunger) and applies mainly to developing countries with histories of chronic hunger and malnutrition. In the European context, however, issues related to over-nutrition (e.g., obesity and chronic disease) are more prominent and is linked to achieving SDG 3 (Good Health and Well-being). Although not explicitly addressed within the SDGs, reducing the use of chemical pesticides in agricultural production works toward attaining SDG 3, specifically Target 3.9 (reducing deaths from hazardous chemicals) as well as SDG 8 (Promoting safe working conditions) (United Nations 2016).

Likewise, the F2F Strategy underlines the connection between developing a sustainable food system and encouraging healthier diets among the EU population. In the Strategy, emphasis is placed on creating a “food environment” that ensures consumers have access to healthy food, as well as information to help them make informed decisions about their food choices. Goals to improve food labeling in terms of nutritional content and production details are intended to facilitate this process (European Commission 2020a). Similarly, Germany’s Sustainable Development Strategy plans to addresses health through delivering better information to consumers via improved labeling and awareness-raising activities that promote healthier diets (Deutsche Bundesregierung 2018).

### *Key digital technologies for achieving sustainability principles*

Our study identified a range of technologies and potential applications for achieving sustainability principles (see Supplementary Table 1). Monitoring enhancing technologies are particularly useful for assessing cross-compliance and designing evidence-based policy (Ehlers et al. 2021). Remote sensing technologies, such as satellite imaging, unmanned aerial vehicles (UAVs), combined with AI can be used to assess changes in land use over large geographic areas (Ferreira et al. 2020), which is useful for monitoring compliance and assessing efficacy of policy (Weersink et al. 2018). Changes in land use can be used as proxies to determine biodiversity conservation, biomass production, and climate change mitigation and adaptation (Weersink et al. 2018). In the future, by combining remote sensing with data obtained from on-farm sensors, digital agriculture could offer real-time and highly granular detail on how production practices are impacting sustainability, such as ecosystem service provisioning, which could open up new avenues for implementing and designing agri-environmental regulations and standards (Ehlers et al. 2021).

Technologies that enhance decision support through increased precision of agrochemical inputs (e.g., variable-rate technologies (VRT), yield monitoring, DSS, GPS tractor navigation, cloud computing) could address a broad range of sustainability principles as outlined in policy by reducing: nitrous oxide emission from fertilizers (climate change mitigation), residual toxicity from pesticides (biodiversity conservation and human health), as well as compaction and nutrient imbalances in soils (soil protection) (Lieder and Schröter-Schlaack 2021).

Communication enhancing technologies can significantly optimize logistics and trade (Poppe et al. 2013, Jouanjean 2019) as well as make food-value chains more transparent to consumers and governments (Walter et al. 2017). Radio-frequency identification (RFID), distributed ledger technologies (i.e., Blockchain), and QR codes enhance traceability of products and transparency on production conditions. In this context, depending on societal demand and legal regulations on food labeling, communication technologies could play a critical part in contributing to the achievement of certain sustainability principles. The growing use of these technologies in the food value-chain implies more importance on behalf of distributors, processors, and retailers on influencing sustainability in future food regimes (Prause et al. 2021).

## Foresight and its implications for sustainability principles and agri-digital law

In this section, the main characteristics of four scenarios as developed by Dönitz et al. (2020) are described in regard to their implications for digital agriculture, agri-digital law, and the achievement of sustainability principles outlined in the previous section. The qualitative scenarios present alternative future framework conditions, influencing functions and requirements of a decision support system for farmers. Although the framework conditions for German agriculture are subject to constant change, these scenarios allow us to make insights about probable future developments, as well as describe political, economic, societal, ecological, and technological developments in order to create robust solutions. These scenarios assist in dealing with the high complexity and interactions of unknown future developments. Using a network of relevant factors, they present a description of possible situations in the future.

### *Description of future scenarios*

To identify the social and technological changes, which are relevant for the agri-business in the upcoming years and over a longer timeframe, it was necessary to look beyond the borders of the sector. Key factors that determine the contexts for the scenarios have been structured using the STEEPL (social, technological, environmental, economic, policy, and legal) approach. According to their relevance for the agri-business, the factors were prioritized and aggregated to 15 key factors with high-relevance (Dönitz et al. 2020). In the current study, to uncover critical factors that are dynamic and strongly linked, the interconnections between the key factors were analyzed (see Section 4.2). The factors “Information flow along the value chain and acceptance of service platforms” and “Diffusion of new technologies in primary production” were identified. Due to their strong influence on and through the other factors, they play an important role in the systems and consequently in the scenarios. They play a special role in the context of the digitalization of agriculture. Therefore, the short descriptions of the scenarios below focus on these two factors.

#### *Scenario 1: Environmental protection by global high tech and regulation; globalized world, government regulation and harmonization*

In Scenario 1, a centralized state control system ensures food supply. An essential point is reduced consumption and the fulfillment of the basic needs. In addition, worldwide coordination of legal standards and global networking works flawlessly. Value chains are transparent and food labels are coordinated. In general, food supply is sustainable and non-profit-oriented. International cooperation provides sufficient momentum to proactively address climate change and to make it a driver for innovation and change. Consumers and industry are open to new technologies. The flow of information along the entire value chain enables consumers to track their products, which puts pressure on producers to maintain high production standards. Most products are purchased through centralized e-commerce managed by the state. Production surpluses flow into a well-functioning global food supply system, ensuring global food security and less food waste. Energy-efficient vertical farming technologies are important for fresh products, like vegetables. Digital platforms assist farmers in their daily work; they provide solutions to various problems.

#### *Scenario 2: Environmental protection by local food circles and qualitative growth; decentralization, diversity and sustainability*

Society recognizes the importance of new technologies in the food industry to assist environmental protection. The direct connection of society to agriculture is enabled not only by the large number and diversity of farms, but also by decentralized retailing acting as information hubs. They control the value chains, make them efficient and transparent, so that labels are no longer necessary. Agricultural production is highly differentiated; the value chains are short and transparent. Consumers accept the seasonal variances of food supply. The communication flow between farmers and consumers is very high. The so-called hybrid farms, a mixture of manually operated large machines and small autonomous robots, work hand in hand. Technologies in the first place have to be beneficial for the environment. Otherwise, they are not accepted. The technological change is promoted by the legal framework. It provides security for business investment in the development of new agricultural technologies and is the source of farmers’ and consumers’ trust.

#### *Scenario 3: Event consumption by face-to-face interaction in local food circles; consumption and direct communication*

People buy in shops or at local food markets. Food shopping is considered important and people look forward to it. They enjoy talking to the producer or simply to their neighbor. As a result, e-commerce only occupies a certain market segment. There is no enthusiasm for new technologies in society, which is reflected by the resistance to advanced digitalization in many areas. Society does not trust or accept new technologies and digital platforms due to security problems in the past and the growing power of global companies. Farmers do not want to rely on new technologies either. They primarily use large, manually driven machines and technological development focuses on assistance systems. Only parts of the agricultural processes have been digitalized, and the connection to further steps in the value chain is missing. Some parts in the production chain have a certain level of intelligence, but there is no connection between them.

#### *Scenario 4: Reduced consumption and de-growth by necessity; growing retail business, no transparence and global food system*

In this scenario, the retail business is the big winner in the global food systems. The area for agricultural production and the area for promoting biodiversity are strictly separated. Both areas are controlled via Agriculture 4.0 with sensors, drones, and other monitoring systems. All this leads to a highly intensive agricultural specialization. New technologies based on AI support farmers in achieving the highest possible efficiency. The data exchange required to optimize AI technologies is not subject to any regulatory restrictions. Retailers are also using AI technologies to design centralized e-commerce that maximizes profit. They exercise high control over agricultural production. The global value chain is not transparent to the consumer. The profit margin for farmers is low and only very specialized farms can economically survive. It is not a high quality but a low price that matters to consumers. Extreme weather conditions challenge agricultural production. Farmers’ cooperation is assisted by digital platforms, especially the sharing of large machinery is promoted by that.

### *Combination of scenarios and key technological areas*

The analysis of the interrelationships between key factors assists to reveal main drivers of change. This analysis helped to achieve a common understanding of (i) how the key factors in context scenarios influence each other and—as a consequence—(ii) to shape different context scenarios by identifying the most crucial interrelations of factors. To evaluate the extent of influence between each pair of key factors (in both directions), the following scales have been used: “0”: no direct influence; “1”: medium influence; “2”: strong influence. On this basis different characteristics of each factor, as an element in the system consisted of 15 factors, can be specified: active and passive factors (with high influence or affectability) as well as critical or dormant factors (with high or low involvement in the system) (Vester 2019). Some key factors have a strong influence on all other factors in this system and at the same time were identified to play an important role for digitalization of agriculture. The influence analysis conducted to build the scenarios in the project DAKIS (Dönitz et al. 2020) shows that the factors “Information flow along the value chain and acceptance of service platforms” and “Diffusion of new technologies in primary production” have a strong influence on all other factors considered in scenarios. Therefore, in the following, these two factors are described with regard to their role within the key technological areas in terms of monitoring, decision support, and communication enhancing technologies (see Table 4 and Supplementary Table 2).

**Table 4:** Scenario 1 and 4. The Role of the scenario factors “Information flow along the value chain and acceptance of service platforms” and “Diffusion of new technologies in primary production” within the key technological areas. For complete analysis of all scenarios see Supplementary Table 2

Hotspots of digitalization within the scenarios		Key technological areas		
		Monitoring	Decision support	Communication
Scenario 1	**Accepted platform with seamless information flow** New technologies and the expansion of network coverage allow more people to retrace agricultural production methods. Information is exchanged between producer and costumer.	• high transparency of the value chain encourages monitoring and the further use of the generated data	• consumers are able to retrace their products, which puts pressure on producers to uphold high production standards• efficiency improvements in the whole process from smart production until delivery of goods by connected and verified (blockchain) information, learning effects from big data and just-in-time optimizations	• new technologies and the expansion of network coverage allow more people to have access to knowledge about agricultural production methods• knowledge expansion in all directions: Digital platforms with detailed information about complete production chain • USP for farmers to give detailed information about their production environment (also as a business model)• seamless flow of information between every step of production chain; bidirectional flow of information (from producer to costumer, from costumer to producer)
	**AI farm** Sensors are integrated in every part of the production chain and collect various kind of data. These information enable the use of artificial intelligence at every stage of the value chain.	• sensors are integrated in every part of production and allow a resource efficient management of input flows • sensors on the farm enables the diversified and side specific management of land which directly promotes biodiversity and ecosystems	• there are different application of AI on the farm; widely deployed are small scaled autonomous robotics with advantages for efficiency and safety • the farmer has more diverse business management responsibilities, e.g. AI supports making economic decisions by providing sales figures in order to adjust production	• the AI Farm is very efficient and successful, as information flow along the whole value chain is possible • e-agriculture strategies address ICT opportunities, with the agricultural production as a focal point, but as well integrating the well-connected agricultural production chain
	**Legal consequences of digitalization requirements**			
Scenario 4	**Retailer is the information hub** Retailers have a major influence on prices, quality, product lines and production conditions. AI is used for intelligent pricing and data for customer profiles is collected to maximize profit.	• retailers will have the possibility to monitor production conditions and anticipate the yields • retail companies collect data about their customers to generate customer profiles in combination with other available data; the data can be used for dynamic pricing and individual marketing to maximize profit	• management decisions will be supported by information from the demand side	• data management is in the hand of the retailer• communication is controlled by the retailer and centralized structures prevail • the retailer is the information hub within the value chain • the intensive use of AI offers a wide range of possibilities for retailers who are using production and processing data for intelligent pricing and to adjust customers demand according to food offerings• there is no seamless information flow from producer directly to consumer and from consumer to producer
	**AI farm** Sensors are integrated in every part of the production chain and collect various kind of data. These information enable the use of artificial intelligence at every stage of the value chain.	• sensors are integrated in every part of production and allow a resource efficient management of input flows • sensors on the farm enables the diversified and site-specific management of land which directly promotes biodiversity and ecosystems • data points collected are managed by the retailer, but farmers do have access to make informed management decisions	• there are different application of AI on the farm; widely deployed are small-scale, autonomous robotics with advantages for efficiency and safety • the farmer has more management responsibilities and makes joint decisions with the retailers, as all the information flow is bundled there	• the AI Farm is works very efficient and data flows are directed towards the retailer• e-agriculture strategies are shaped in large part by the retailer
	**Legal consequences of digitalization requirements**	• as retailers will have the possibility to monitor production conditions and anticipate the yields, the legal framework has to guarantee data sovereignty for sensitive operational data of the farmer	• as management decisions will be supported by information from the demand side, the legal framework has to ensure that the importance of demand does not outweigh the constraints of sustainable production	• as communication is controlled by the retailer, the legal framework has to ensure that this unequal power relations over information are not exploited; transparency in data management has to be guaranteed

In Table 4, it is explained what the respective assumptions of the two factors, “Information flow along the value chain and acceptance of service platforms” and “Diffusion of new technologies in primary production” per Scenario 1 and per Scenario 4 imply for the requirements of digitalization in terms of monitoring, decision support, and communication enhancing technologies. Furthermore, where applicable, legal consequences of these digitalization requirements are presented. Please see Supplementary Table 2 for complete analysis of all scenarios.

### *Convergence of sustainability principles and scenarios*

In this sub-section, we analyze implications of the four scenarios from the foresight analysis on digital agriculture and the achievement of sustainability principles identified in the policy analysis. The scenarios provide illustrative and contrasting examples of how digital agriculture technologies could impact sustainability.

Scenario 1 (Environmental Protection by Global High Tech and Regulation; Globalized world, government regulation and harmonization) has a high potential for achieving a broad spectrum of sustainability goals through state control and leveraging digital technologies. In this scenario, sustainability goals, as dictated by governments, are consistently achieved with aid of technologies that enhance monitoring, decision support, and communication. For example, communication enhancing technologies (e.g., blockchain, RFID, QR codes) ensure high transparency to consumers on production conditions in terms of their impacts on the sustainability principles of human health, biodiversity conservation, and climate change. Additionally, these technologies as well as on-farm data obtained from management enhancing technologies are used by governments for monitoring compliance with regulations and standards. In terms of primary production, in Scenario 1, technologies that enhance decision support (e.g., sensors, DSS, UAV, VRT, AI, robotics) are wide-spread, significantly reducing the use of pesticides and fertilizers that are harmful to humans and the environment, thereby contributing to the principles of biodiversity conservation, soil protection, climate change mitigation, and human health. Additionally, monitoring technologies (e.g., satellite imaging, agricultural census data) combined with the free flow of harmonized data allow governments to assess whether sustainability goals are being met and to design policy accordingly.

Scenario 2 (Environmental Protection by Local Food Circles and Qualitative Growth; Decentralization, diversity and sustainability) also describe a future food system with a high-degree of digitalization in terms of utilizing monitoring, decision support, and communication enhancing technologies. However, a key distinction of this scenario is that the information flow of food system data is not controlled centrally by state governments, as in Scenario 1, but instead is controlled through decentralized networks of retailers. Here, communication enhancing technologies that promote transparency of productions conditions combined with consumer demand for “greener” products are the main drivers behind achieving sustainability principles. Given this, along with the decentralized and local food system as described by Scenario 2, digital agriculture is most likely leveraged for achieving region-specific goals, meaning that goals formulated at higher policy levels may be less in focus. This could have positive impacts for sustainability principles such as biodiversity conservation, climate change *adaptation*, and soil protection, which generally require site-specific solutions, but negative impacts for principles of climate change *mitigation* and biomass production (e.g., food security), which are primarily addressed by national and international policy and where the momentum of a joint international approach is needed to be effective.

Scenario 3 (Event Consumption by Face-to-Face Interaction in Local Food Circles; Consumption and direct communication) describes a future that is least optimistic in terms of leveraging digital agriculture to achieve sustainability goals. In this scenario, acceptance of digital technologies by farmers and consumers is low due to elevated concerns for data privacy and distrust of large agricultural-tech companies, preferring instead conventional technologies, such as manually driven tractors, and face-to-face communication, such as farmers markets. A lack of consumer preference for healthier and environmentally friendly products means that digital technologies that enhance transparency to consumers are not valued or utilized to their fullest extent. Continuation of conventional management methods means that biomass production is not significantly increased, climate change mitigation is not addressed, soil and biodiversity conditions continue to deteriorate, and lack of systemic monitoring impedes the assessment of policy goal attainment.

Scenario 4 (Reduced Consumption and De-growth by Necessity; Growing retail business, no transparence and global food system) describes a highly digitalized agri-food system controlled by retailers that is similar to Scenario 2. In Scenario 4, however, information flow is completely controlled by international retailers and is not transparent to consumers or governments. Further, a high level competitiveness between service providers means that there is no interportability of farm-generated data. This has important implications for achieving sustainability principles. For example, since production conditions are not transparent, consumer demand for environmentally friendly products cannot be fully realized and government monitoring of farm-level compliance with environmental regulation is impeded. In effect, whether or not sustainability goals are achieved in this scenario is highly subject to the economic interests of retailers and digital technology providers.

## Agri-digital law

The function of law consists of realizing the worked out sustainability goals and by setting clear rules. This provides clear rules for transactions between stakeholders of digitally driven farming systems that balance the legitimate interests of farmers on the protection of their personal/entrepreneurial data and the interest of service providers to run new business models (Härtel 2019). Additionally, an Agri-Digital Law, as developed by Ines Härtel, is able to give incentives for investments in the development and use of innovative technologies, as outlined in the developed foresight scenarios. At the current stage, a holistic legal framework does not exist and there are still many legal questions to clarify that depend on the technical design of ICT-based applications and devices in general. However, some groundwork has been established which gives orientation for the further design of a future legal framework (Härtel 2019) and which could be interpreted as a first step into the realization at least of the first and second scenario mentioned above, i.e., environmental protection by global high tech and regulation on the one hand, and environmental protection by local food cycles, qualitative growth, decentralization, and diversity on the other hand.

In a general manner, a tendency for a digital transformation in agriculture can be clearly identified. The European Commission’s draft amendments to the Common Agricultural Policy are steering into this direction. Article 13 COM(2018) 392 final, for example, stipulates that “Agricultural Knowledge and Innovation Systems” should integrate technological and scientific information for the benefit of agriculture. It is thus paradigmatically assumed that digitalization should contribute to increasing sustainability effects. This would tend to speak in favor of the first two scenarios, in which the use of digitally driven technologies in agriculture is assumed. The same applies to the Arable Farming Strategy 2035, which envisages the creation of legal framework conditions for the use of digital technologies, especially for autonomous driving land machines, as a measure.

Regardless of the degree of digitalization of the agricultural sector, the basic principles of the General Data Protection Regulation (GDPR) have to be taken into account in the area of communication, which subject data exchange processes to legal regulations if a personal reference to the transferred data can be established. In this respect, technological implications also arise for monitoring. In this context, the politically articulated data sovereignty of farmers within the framework of the Arable Farming Strategy 2035 must be taken into account, as it is currently realized in particular through the basic provisions of the GDPR. According to the case law of the European Court of Justice in the Schecke case, the GDPR applies to the majority of agricultural businesses, as the company name allows conclusions to be drawn about the natural persons behind it, particularly in the case of smaller agricultural businesses (Kipker and Bruns 2020). This lays an important foundation for farmers’ data sovereignty, which is also relevant from a technological point of view with regard to monitoring. Furthermore, a “Code of conduct on agricultural data sharing by contractual agreement” has been in place at European level since 2018 and has been signed by a total of nine stakeholder organizations (Härtel 2020b). At the German national level, there is also a scientific recommendation on “farmer data sovereignty” in the context of an agricultural data space with “agricultural data” as a new category of data (Härtel 2020b).

Portability of non-personal data files is subject to self-regulation under Regulation (EU) 2018/1807 on a framework for the free movement of non-personal data in the European Union. According to this, the service providers are to develop their own rules of action within a framework predetermined by EU law (Härtel 2020b). In this respect, for example, certification systems are to be established to enable users to benchmark data processing products. Environmental management could also be able to be taken into account here. With regard to the portability of data, the Act against Restraints of Competition was recently amended, according to which Section 19a (2) No. 5 the Federal Cartel Office is authorized to intervene by way of platform supervision against interoperability restrictions that hinder competition. The question of the compatibility of this regulation with European Union law is viewed critically in the literature (Grünwald 2020).

With regard to on-farm management, the first legal foundations for the use of distributed ledger technologies are developing (i.e., Blockchain), which in turn can increase the validity of the data input for farm management systems. From a legal perspective, the use of distributed ledger technologies in the context of management systems must take into account that the interest in valid and high-quality data must be appropriately balanced with the right to be forgotten from Article 17 of the GDPR (Schöbel 2021).

If future IT-supported management decisions takes the demand side into account and the retailer thus functions as an information hub, it is possible to fall back on an already quite differentiated regulatory regime which, in addition to special legal regulations, such as those for organic products, regional products, or marketing standards of the Common Agricultural Policy, is based in particular on the EU Food Information Regulation and specific legal provisions at the European and nationals levels.

In case that future data platforms, such as those on which a preliminary study in 2020 was based (Bartels et al. 2020) and digital decision support systems pave the way toward an AI or hybrid farm (as outlined in Scenarios 1 and 2), the legal implications of the use of AI systems in the backend would have to be taken into account (Härtel 2020a). To date, the use of artificial intelligence in agriculture is not subject to special legal safety requirements. However, the general liability regime is already applicable, consisting of the General Product Safety Directive, the Machinery Directive implemented in Germany by the 9th Regulations to the Product Safety Act, and the Regulation on the Approval and Market Surveillance of Agricultural and Forestry Vehicles (Härtel 2020a). In April 2021, the European Commission released a proposal for an Artificial Intelligence Act. This regulation proposal implies a differentiated statutory regime which contains increased legal requirements for the use of AI in critical infrastructures. Since agriculture ensures food security for the population, it has to be regarded as a part of the critical infrastructure. In this respect, instrumental provisions are made for risk management systems, quality requirements for training, validation and test data sets, technical documentation regarding risk classification, monitoring obligations throughout the life cycle, transparency of information to users, supervision by natural persons, accuracy, robustness, as well as cybersecurity of the AI system.

In the run-up to any technological implementation of platform and decision support systems, the limits of agricultural digital law must also be taken into account, which arise, for example, for the use of drone-based sensor technologies or the use of robots (Härtel 2019). A high degree of legal innovation is apparent in the Commission’s draft of the planned Digital Services Act, which is to contain a comprehensive regulatory concept for digital services in the future.

The possible future use of digitally driven technologies requires stakeholder trust, which in turn depends heavily on cybersecurity. With regard to cybersecurity, ENISA (European Union Agency for Cybersecurity) is to act as a networking body between member state authorities, thereby contributing to ensuring a high level of security of network and information systems, which is the regulatory subject of Regulation (EU) 2019/881 on ENISA and on information and communications technology cybersecurity certification as well as of Directive (EU) 2016/1148 concerning measures for a high common level of security of network and information systems across the Union (Specht 2018).

All the aforementioned groundwork has to be further developed, but from legal perspective a clear tendency toward the digital transformation of agriculture can be observed. This expressively implies systems for the exchange of agricultural knowledge and information. If technical evolution and legal design go hand in hand, then the conditions for the formulation of a suitable, coherent, and consistent legal framework are necessary. With regard to the future, legal experts should work together in an interdisciplinary fashion in order to be able to advise legal policy concerning the future development of a legal framework that leads to a balance of interests, gives incentives for innovation and is adaptive for disruptive technologies that might arise within the context of digital transformation of agriculture.

## Discussion

Digital agriculture could potentially deliver improvements to sustainability across food systems. This stance can be found in several of the policies we reviewed, although only to a limited extent. Of the reviewed policies, the F2F Strategy, 2035 Arable Farming Strategy, and the National Bioeconomy Strategy stood out in terms of their incorporation of digital agriculture in their documents. Our study showed that policies consider digital agriculture mostly in terms of resource use efficiency, while its benefits for achieving other sustainability principles such as biodiversity conservation, soil protection, and climate change adaptation and mitigation are not thoroughly reflected. Similar findings of policy from high-level institutions were found by Lajoie-O’Malley et al. (2020). Nevertheless, the reviewed policies converged on certain points concerning frame conditions for implementing digital agriculture, such as developing a statutory framework, working toward data harmonization, as well as increasing high speed internet availability to rural areas.

Our study showed technologies that improve decision support, such as VRT, cloud computing, IoT, yield mapping, digital soil mapping, sensors, and UAVs are particularly relevant toward achieving the majority of agriculture-related goals and, by extension, diverse sustainability principles. Further, technologies that enhance monitoring such as satellite imaging, AI, and agricultural census data are particularly relevant for promoting biomass production, climate change mitigation and adaption, as well as biodiversity conservation. Lastly, communication technologies such as RFIDs, QR codes, and distributed ledger technology (e.g., Blockchain) promote transparency along the value chain, thereby contributing to goals related to health and nutrition as well as biodiversity conservation. Given the rapid growth and innovation in digital agriculture, policy should do more to highlight these potential applications and refer to the burgeoning literature on the topic.

A shortcoming of the reviewed policies should be noted. Although they acknowledge the growing role of consumer demand in shaping agricultural production systems, they do not sufficiently consider how digitalization is increasingly embedded in this process. Our foresight analysis suggests that communication enhancing technologies will bring consumers and producers closer together, which is also echoed in the literature (Birner et al. 2021). Digital technologies can provide more detail and transparency to consumers on the production conditions and nutritional content of their food, as well as provide farmers with better information on consumer preferences and trends. This new dynamic between consumer and producers could be a decisive factor in achieving sustainability goals in the future. In this context, our study also shows a growing importance of retailers in the agri-food sector as brokers of information between farmers and consumers, potentially connecting them in short value chains, which means retailers could have significant influence over agriculture in future food regimes (Prause et al. 2021). Failure of policy to recognize how digitalization is transforming food value chains could be a shortcoming in connecting the farm to fork and considering the agri-food sector as a whole.

There are many adoption barriers of digital agriculture technologies. High investment costs (Rose and Chilvers 2018) and lack of training and advisory services for farmers are some of the main barriers to adoption, especially for small- and medium-scale farmers (Paustian and Theuvsen 2017). Policy can help surmount these barriers through offering financial assistance to farmers and innovators in the form of tax-breaks and/or subsidies that help compensate short-term opportunity costs and long-term financial risks associated with technological innovation and investment (Ehlers et al. 2021). As suggested in the literature and taken up in the 2035 Arable Farming Strategy and the F2F Strategy, providing agriculture training (i.e., digital skill sets) and advisory services for farmers could foster a more inclusive digital agriculture for small-scale, agri-food businesses (Piñeiro et al. 2020; Long et al. 2016). Coupling advisory services and training with financial assistance for digital technologies will increase chances that digital agriculture will be leveraged to its fullest potential. Ultimately, these type of measures may help to avoid a digital divide between large-scale and small-scale farmers in the future (Rotz et al. 2019b; Revenko and Revenko 2019).

There are many things to account for in regard to how ownership of data will shape the future of digital agriculture. As data becomes more central in the future agri-food sector, whoever controls this data will have immense influence on dictating to which ends it is being used, including how and if it used for achieving sustainability principles. Currently, there is a trend toward consolidation of the control of data among large agriculture technology companies (i.e., “data grab”), which raises doubts as to whether digital agriculture when left in the hands of big business will be used for sustainable ends or reinforce neoliberal and productivist paradigms (Birner et al. 2021; Prause et al. 2021; Clapp and Ruder 2020). With the exception of the 2035 Arable Farming Strategy, which mentions the need to create a statutory framework and explore preconditions of data sovereignty, there is a striking absence of language addressing this issue in the reviewed policies.

Although fragmented, the current framework of laws surrounding digital agriculture is evolving, albeit at a pace behind technological development. Precedence shows that laws are typically reactive. This puts policy in a position of responsibility that should anticipate the digital transformation of agriculture and be an active guide toward steering it in the direction of sustainability. It is the view of these authors that this can best be achieved by empowering farmers’ enterprises by protecting their legal rights in regard to the control of their data (i.e., data sovereignty). The literature provides many examples of ways for leveling the playing field between large ag-tech companies and farmers. Overall, it may be necessary to reconceptualize data generated from agriculture as its own class of data with its own set of regulations (Härtel 2020b). This would be a key step in making certain types of non-personal data available to farmers, companies, as well as government and research institutions who could use it to achieve wider sustainability objectives.

We would like to mention a few limitations of this study. Our review of policies is not exhaustive, meaning that some policies that consider digital agriculture may be missing from our analysis such as the Common Agriculture Policy of the EU, for example, which would be too wide for the scope for this paper and would deserve a study in its own right. However, our review does not intend to be exhaustive, but rather to demonstrate how current and widely recognized sustainability policies potentially intersect with digital agriculture. In future studies, it would certainly be interesting to look at which policy measures can be used not only at the farming level, as described above, but also, for example, at the level of consumers or retailers. Additionally, by focusing on Germany, we were able to provide an in-depth analysis. However, depending on the country of interest, policy development and the digital transformation of agriculture may look very different. This means future research should focus on how policy and digital agriculture is taking shape in other countries to assess and compare impacts of digitalization under different frame conditions (see Fleming et al. 2021).

In regard to the scenarios, although they provide rich details on probable futures, they are theoretical propositions about what *could* happen in the future. The inherent uncertainty of the future makes it impossible to anticipate all factors that might have an influence on digital agriculture, especially considering the rapid changes brought about by digital technologies in other parts of society. Indeed, an unforeseeable event could alter the validity of our scenarios. Nevertheless, it is prudent to make assumptions about probable futures in order to anticipate potential change and avoid sub-optimal outcomes. Finally, in regard to our legal analysis, there are surely many developments of statues in private law that will shape digital agriculture, but these would be impractical to cover within the scope of this research. However, we find that the precedent established by public law more relevant to the level of analysis of this study.

## Conclusion

Whether or not digital agriculture can provide solutions to sustainability problems depends on how it is currently embedded in policy, as well as how future frame conditions and legal settings shape its implementation. Otherwise, digitalization may just become another instrument for reinforcing the paradigm of economic efficiency. Research is therefore required that takes stock of missions and goals of current societal sustainability imperatives that potentially intersect with digital agriculture, while identifying optimal future and legal frame conditions for exploiting the potential of digitalization in order to achieve societal targets. In so doing, such research will facilitate the development of mission-oriented policies that contemplate and anticipate the institutional and technological preconditions and potential unintended consequences of evolving technological transition pathways (Klerkx and Rose 2020).

In this regard, our study offers a unique perspective on how digital agriculture may be leveraged to achieve policy targets under different future scenarios and an evolving legal framework. The results show that digital agriculture is taken up in some high-level policies, but only to a limited extent. However, we identified how digital technologies could be applied more broadly in agri-food systems to achieve sustainability principles outlined in policy strategies. Additionally, our results corroborated those found in the literature that the adoption of digital technologies and the ends to which they are being used are largely dependent on future data ownership regimes. Our foresight analysis highlighted how control of information and ownership of data may unfold under different probable futures and what this means for the achievement of sustainability principles. The legal analysis provided additional insights to a preliminary, fragmented legal framework that is currently evolving in favor of free flow of non-personal, farm-generated data for public and private use.

Overall, the integration of monitoring, decision support, and communication enhancing technologies along the entire agri-food chain is needed to cultivate a real “game changing” Agricultural 4.0. It is therefore prudent of high-level policy to be future-oriented by anticipating a greater role of digitalization not only in agricultural production, but also in governance, retail, and consumption. This will probably require a change in thinking about agriculture, since digitization may shift or blur traditional lines in agri-food systems, bringing us in new ways closer to the food we eat.

## Supplementary Information

ESM 1 (DOCX 16 kb)

ESM 2 (DOCX 18 kb)

## Data Availability

All articles reviewed are available online.
