# Supplementary material for: Future agricultural systems and the role of digitalization for achieving sustainability goals. A review
Source: Agron Sustain Dev. 2022 Jul 6;42(4):70. doi: 10.1007/s13593-022-00792-6 (PMC9258761; doi:10.1007/s13593-022-00792-6)
Supplement: Supplementary file 1 — (DOCX 16 kb) [file 13593_2022_792_MOESM1_ESM.docx]

| **Supplementary Table 1. Cross-cutting agricultural sustainability principles and related strategy goals with implicit links to key enabling technologies** | | | | | |
| --- | --- | --- | --- | --- | --- |
|  |  |  | **Key enabling technologies** | | |
| **Sustainability principle** | **Policy** | **Goals** | **Monitoring** | **Decision support** | **Communication** |
| Biomass production | SDGs | Zero hunger (SDG2) and production of bio-materials/bio-fuels (SDG 7.2) | Artificial intelligence (AI), climate data, market information, agricultural census data | yield mapping, unmanned aerial vehicle (UAV), Internet of Things (IOT), variable rate technology (VRT), decision support system (DSS), GPS tractor navigation |  |
|  | F2F Strategy | promote resilience of food systems to external shocks and crises and strengthen bioeconomy | AI, climate data, market information, agricultural census data | yield mapping, unmanned aerial vehicles, IoT, VRT, DSS, GPS tractor navigation |  |
|  | Sustainable Development Strategy | contribute to world food basket | AI, yield data, market information, agricultural census data | yield mapping, UAV, IoT, VRT, DSS, GPS tractor navigation |  |
|  | Bioeconomy Strategy | enhance biological knowledge and establish raw material base for industry | AI, yield data, market information, agricultural census data |  |  |
| Climate change mitigation and adaptation | Paris Agreement | Limit emission via INDCs | agricultural census data, AI, satellite imaging |  |  |
|  | SDGs | develop long-term mitigation and adaptation strategies | agricultural census data, AI, satellite imaging |  |  |
|  | F2F Strategy | reduce livestock emissions through innovative feed additives | agricultural census data | biometiric sensing, predictive feed intake models |  |
|  |  | promote soils as carbon sinks | digital soil mapping | digital soil mapping, DSS |  |
|  | National Climate Action Plan | reduce nitrous oxide emissions from fertilizers (70kg N/ha) by 2032 |  | VRT, UAV, DSS |  |
| Biodiversity conservation | SDGs | maintain ecosystem integrity | UAV, satellite imaging | DSS |  |
|  |  | genetic diversity |  |  |  |
|  | F2F strategy | reduce pesticides by 50% by 2030 |  | VRT, UAV, IoT, DSS, GPS tractor navigation |  |
|  |  | reduce fertilizers by 20% by 2030 |  | VRT, UAV, IoT, DSS, GPS tractor navigation |  |
|  | National Sustainable Development Strategy | increase landscape diversity and quality | UAV, satellite imaging |  |  |
|  |  | promote organic farming |  | DSS | Radio-frequency identification (RFID), QR code, Blockchain |
|  |  | reducing agricultural inputs such and nitrogen and phosphorous |  | VRT, UAV, IoT, DSS, GPS tractor navigation |  |
|  | 2035 Arable Land Strategy | promoting habitat diversity and connectivity at the landscape level | UAV, satellite imaging |  |  |
|  |  | establishing regional goals and monitoring mechanism | satellite imaging, UAV |  |  |
|  |  | evaluating economic impacts of biodiversity measures | agricultural census data, AI, satellite imaging |  |  |
| Soil protection | SDGs | restore degraded land and soil | digital soil mapping | VRT, GPS tractor navigation, UAV, small-scale automated tractors, DSS, IoT |  |
|  |  | utilizing agricultural production methods that improve land and soil quality |  | VRT, UAV, IoT, small-scale automated tractors, DSS |  |
|  | F2F Strategy | reduce pesticides by 50% by 2030 |  | VRT, GPS tractor navigation , UAV, DSS, IoT |  |
|  |  | reduce fertilizers by 20% by 2030 |  | VRT, GPS tractor navigation , UAV, DSS, IoT |  |
|  | National Bioeconomy Strategy | systemic and location-specific approach for the production of biogenic resources | digital soil mapping, AI, yield data, market information, agricultural census data | VRT, GPS tractor navigation , UAVs DSS, IoT |  |
|  | 2035 Arable Land Strategy | promote soil fertility and soil biodiversity | digital soil mapping, AI, yield data, market information, agricultural census data | VRT, GPS tractor navigation , UAV, DSS, IoT |  |
|  |  | reduce erosion and compaction |  | Soil sensors, GPS tractor navigation, small-scale automated tractors |  |
|  |  | humus content should be kept stable through admixture |  |  |  |
|  |  | land take by non-agricultural usage is to be reduced to under 30 ha per day and net zero by 2050 | satellite imaging |  |  |
| Human health | SDGs | promote health and well-being (SDG 3) |  |  | RFID, QR code, Blockchain |
|  |  | promote safe working conditions |  | VRT, GPS tractor navigation, UAVs, small-scale automated tractors, DSS, IoT |  |
|  |  | reduce deaths from hazardous chemicals |  | VRT, GPS tractor navigation, UAVs, small-scale automated tractors, DSS, IoT |  |
|  | F2F Strategy | promote healthier and greener diets through improved labelling of nutrition content and production details |  |  | RFID, QR code, Blockchain |
|  | Sustainable Development Strategy | Promoting healthier diets through improved labelling |  |  | RFID, QR code, Blockchain |
