# Supplementary material for: Future agricultural systems and the role of digitalization for achieving sustainability goals. A review
Source: Agron Sustain Dev. 2022 Jul 6;42(4):70. doi: 10.1007/s13593-022-00792-6 (PMC9258761; doi:10.1007/s13593-022-00792-6)
Supplement: Supplementary file 2 — (DOCX 18 kb) [file 13593_2022_792_MOESM2_ESM.docx]

**Supplementary Table 2: The Role of the scenario factors “Information flow along the value chain and acceptance of service platforms” and “Diffusion of new technologies in primary production” within the key technological areas**

|  |  | **Key technological areas** | | |
| --- | --- | --- | --- | --- |
|  | **Hotspots of digitalization within the scenarios** | **Communication** | **Decision support** | **Monitoring** |
| Scenario 1 | **ACCEPTED PLATFORM WITH SEAMLESS INFORMATION FLOW** New technologies and the expansion of network coverage allow more people to retrace agricultural production methods. Information is exchanged between producer and costumer. | • new technologies and the expansion of network coverage allow more people to have access to knowledge about agricultural production methods • knowledge expansion in all directions: Digital platforms with detailed information about complete production chain  • USP for farmers to give detailed information about their production environment (also as a business model) • seamless flow of information between every step of production chain; bidirectional flow of information (from producer to costumer, from costumer to producer) | • consumers are able to retrace their products, which puts pressure on producers to uphold high production standards • efficiency improvements in the whole process from smart production until delivery of goods by connected and verified (blockchain) information, learning effects from big data and just-in-time optimizations | • high transparency of the value chain encourages monitoring and the further use of the generated data |
|  | **AI FARM** Sensors are integrated in every part of the production chain and collect various kind of data. These information enable the use of artificial intelligence at every stage of the value chain. | • the AI Farm is very efficient and successful, as information flow along the whole value chain is possible  • e-agriculture strategies address ICT opportunities, with the agricultural production as a focal point, but as well integrating the well-connected agricultural production chain | • there are different application of AI on the farm; widely deployed are small scaled autonomous robotics with advantages for efficiency and safety  • the farmer has more diverse business management responsibilities, e.g. AI supports making economic decisions by providing sales figures in order to adjust production | • sensors are integrated in every part of production and allow a resource efficient management of input flows  • sensors on the farm enables the diversified and side specific management of land which directly promotes biodiversity and ecosystems |
|  | **Legal consequences of digitalization requirements** |  |  |  |
| Scenario 2 | **RETAILER IS THE INFORMATION HUB** Retailers have a major influence on prices, quality, product lines and production conditions. AI is used for intelligent pricing and data for customer profiles is collected to maximize profit. | • data management is in the hand of the local retailers • there is a high need of communication on the local level; decentralized structures have to be created to enable communication on the small scale • communication is controlled by the local retailer • the retailer is the information hub within the local and diversified value chain  • the intensive use of AI offers a wide range of possibilities for retailers who are using production and processing data for intelligent pricing, to adjust customers demand according to the food offer • there is no seamless information flow from producer directly to consumer and from consumer to producer | • management decisions will be supported by information from the demand side  • local requirements from the consumer side can be brought together with local production conditions | • retailers will have the possibility to monitor production conditions on the local level and anticipate the yields  • retail companies collect data about their customers to generate customer profiles in combination with other available data. The data can be used for dynamic pricing and individual marketing to maximize profit. Region specific conditions can be taken care of. |
|  | **HYBRID FARM** There is a mixture of large, manually operated machines and small autonomous robots capable of tracking factors such as soil moisture, plant health and animal diseases. Effective sensors are available and used for the partly-smart food value chain. | • effective sensors are available and used for specific parts of the food value chain; this means only parts of value chain are smart; still, there is communication along the value chain.  • the communication flow between the different forms of machinery has to be guaranteed | • mixture of large machines (operated manually) and small autonomous robots • precision agriculture is used to reduce the environmental footprint | • Sensors are mounted on robots that automatically travel around the farm and are capable of tracking factors such as soil moisture levels, monitoring plant health and detecting animal disease such as cow mastitis. • With apps farmers can track the real-time development of their crops from a computer or mobile phone |
|  | **Legal consequences of digitalization requirements** | • as communication is controlled by the local retailer, the legal framework has to ensure transparency in data management | • as management decisions will be supported by information from the demand side, the legal framework has to ensure that the importance of demand does not outweigh the constraints of sustainable production | • as the retailers will have the possibility to monitor production conditions on the local level and anticipate the yields, the legal framework has to guarantee data sovereignty for sensitive operational data of the farmer |
| Scenario 3 | **NO ACCEPTANCE OF DIGITAL PLATFORM BUT RICH ANALOG INFORMATION**  There is a resistance to digitalization and privacy loss is not accepted. Costumers shop locally regional food and exchange information at the point of sale. | • there is no acceptance of privacy loss and data exchange is very restricted  • shopping local food and high information exchange at the point of sale, instead of using digital platforms • there is no acceptance of digital platform but rich analog information | • there is a resistance to digitalization and low trust for digital technologies caused by security problems and the growing power of global corporations | • few monitoring activities due to obstacles of using collected data |
|  | **MACHINE FARM** Only parts of the farming process are digitalized, most processes are still analog. There are manually driven huge machines for larger fields and assistant systems are being developed. The different production steps are smart, however there is no connection in between. | • there is little communication because the value chain is not connected  • there is no communication between the different parts of the machinery | • manually driven huge machines and large fields • technological development only for assistant systems • trust in external human advisors | only parts of the farming process are digitalized and the majority of processes is still analog.  • there is some intelligence within the production chain but no connection in-between, leading to some small, smart islands within the complete production chain |
|  | **Legal consequences of digitalization requirements** |  | • as there is trust in external human advisors, digitalization assists to manage huge fields, but farmers rather trust personal advice than a decision support tool; therefore the legal framework has to support this individual cooperation between the farmer and the advisor | • as only parts of the farming process are digitalized, monitoring is not evenly distributed, because it cannot playout its major advantages if only parts of the value chain are connected and the information flow is interrupted, therefore a legal framework could support data sharing in some technological areas |
| Scenario 4 | **RETAILER IS THE INFORMATION HUB** Retailers have a major influence on prices, quality, product lines and production conditions. AI is used for intelligent pricing and data for customer profiles is collected to maximize profit. | • data management is in the hand of the retailer • communication is controlled by the retailer and centralized structures prevail  • the retailer is the information hub within the value chain  • the intensive use of AI offers a wide range of possibilities for retailers who are using production and processing data for intelligent pricing and to adjust customers demand according to food offerings • there is no seamless information flow from producer directly to consumer and from consumer to producer | • management decisions will be supported by information from the demand side | • retailers will have the possibility to monitor production conditions and anticipate the yields  • retail companies collect data about their customers to generate customer profiles in combination with other available data; the data can be used for dynamic pricing and individual marketing to maximize profit |
|  | **AI FARM** Sensors are integrated in every part of the production chain and collect various kind of data. These information enable the use of artificial intelligence at every stage of the value chain. | • the AI Farm is works very efficient and data flows are directed towards the retailer • e-agriculture strategies are shaped in large part by the retailer | • there are different application of AI on the farm; widely deployed are small-scale, autonomous robotics with advantages for efficiency and safety  • the farmer has more management responsibilities and makes joint decisions with the retailers, as all the information flow is bundled there | • sensors are integrated in every part of production and allow a resource efficient management of input flows  • sensors on the farm enables the diversified and site-specific management of land which directly promotes biodiversity and ecosystems  • data points collected are managed by the retailer, but farmers do have access to make informed management decisions |
|  | **Legal consequences of digitalization requirements** | • as communication is controlled by the retailer, the legal framework has to ensure that this unequal power relations over information are not exploited; transparency in data management has to be guaranteed | • as management decisions will be supported by information from the demand side, the legal framework has to ensure that the importance of demand does not outweigh the constraints of sustainable production | • as retailers will have the possibility to monitor production conditions and anticipate the yields, the legal framework has to guarantee data sovereignty for sensitive operational data of the farmer |
